# Supplementary material for: Age influences the thermal suitability of Plasmodium falciparum transmission in the Asian malaria vector Anopheles stephensi
Source: Proc Biol Sci. 2020 Jul 22;287(1931):20201093. doi: 10.1098/rspb.2020.1093 (PMC7423674; doi:10.1098/rspb.2020.1093)
Supplement: Supplementary Information [file rspb20201093supp1.pdf]

Supplemental Information

Age influences the thermal suitability of *Plasmodium falciparum* transmission in the Asian malaria vector *Anopheles stephensi*

Miazgowicz, KL, Shocket, MS, Ryan, SJ, Villena OC, Hall, RJ, Owen, J , Adanlawo, T, Balaji, K, and Johnson, LR, Mordecai, EA, Murdock CC

Proceedings of the Royal Society Biological Sciences

http://doi.org/10.1098/rspb.2020.1093

Methods

Rearing of Anopheles stephensi

We sourced a long-standing colony (~40 years) of *An. stephensi* mosquitoes from Pennsylvania State University which were originally obtained from the Walter Reed Army Institute of Research (Silver Spring, MD, USA). The *An. stephensi* colony was consistently held at standard insectary conditions (27°C ± 0.5°C, 80% ± 5% relative humidity, and a 12L:12D photoperiod) prior to starting the adult life history experiment. We hatched and transferred 110 larvae to plastic trays (6 Qt., 12.4 cm x 34.6 cm x 21.0 cm) containing 500 mL of distilled water and maintained on a daily regimen of 100 mg ground TetraMin fish flakes. To ensure age-matched individuals were used in the life table experiment, only pupae present on day 9 post-hatch (peak pupal stage) were collected and placed into adult cages. Any pupae remaining after 24 hr were removed. We provided adult mosquitoes with a solution of 5% dextrose and 0.05% para-amino benzoic acid (PABA) upon emergence. Given the extended duration of these experiments (~60 days), multiple blood donors were used throughout each replicate. For colony maintenance, *An. stephensi* were fed whole human blood (O+, healthy male < 30 years, Interstate Blood Bank, TN, USA).

Statistical analyses (expanded)

All statistical analyses were performed using R (version 3.4.1) (1). We used generalized linear mixed models (GLMM) R package <*lme4::glmer()* (2) > to estimate the effects of temperature, mosquito age, and their interaction on the proportion of females that imbibed blood on a given day (i.e., the number of females that

took a blood meal on a given day out of the total number of females alive on that day for each temperature treatment) and the mean daily egg production (i.e., the number of eggs laid on a given day divided by the total number of females alive on that day in a given temperature treatment). Temperature, age, and their interaction were included as fixed effects. Both linear and non-linear terms for temperature and age were included as continuous variables that were scaled and centered. Random factors initially included block, blood donor, and individual as categorical variables. We used minimum Akaike information criterion (AIC) values (3) to compare and select our final models (**SI_Table 1, SI_Table 2, SI_Table 3**).

We also used a Log-rank test with R package <*survival::survdiff()* (4)> on Kaplan-Meier estimates to determine if survivorship differed with temperature. Lastly, to determine if the daily survival rate changed across the lifespan of the mosquito, we fit a variety of survival distributions, which allow either for a constant (exponential) or variable daily mortality rate (log-normal, gamma, Gompertz, and Weibull) with R package <*flexsurv* (5)> to the Kaplan-Meier estimates. AIC values were used to choose between the candidate models. In addition, survival distributions were fit to the Kaplan-Meier estimates from each temperature treatment independently to confirm the best-fitting survival distribution did not vary with temperature (**SI_Table 4)**.

Derivation of relative* R_0(T) *models

The Ross-Macdonald expression is commonly used to represent R_0, the basic reproductive number, or the number of secondary cases expected to arise from a primary infection given a fully susceptible population (**SI_Equation 1**) (6).

$$R_0 = \sqrt{\frac{Ma^2bce^{-\mu EIP}}{Nr\mu}} \quad \textbf{(SI\_Equation 1)}$$

R_0 is composed of parameters for vector abundance, *M*, human host abundance, *N*, the vector biting rate, *a*, vector competence, *bc,* or the product of the proportion of vectors that become infected (*b*) and the proportion of human hosts that become infected (*c*), the vector daily mortality rate, μ, human recovery rate, *r,* and the extrinsic incubation period of the pathogen (*EIP*). In accordance with previous studies, we incorporate how these traits are affected by temperature. First, as previously (7), the effect of temperature (*T*) and rainfall (*R*) on mosquito density (*M*) was accounted for by including temperature-sensitivity to mosquito life history traits such

as per capita birth rate (λ), per capita death rate (μ), daily survival probability of larval (p_L), larval development time (τ_L) and the effect of rainfall on as per capita birth rate (λ), daily survival probabilities of eggs (p_E), larvae (p_L), and pupae (p_P) as shown in **SI_Equation 2**.

$$M(R,T) = \frac{\lambda(R,T)}{\mu(T)}\ ;\ \lambda(R,T) = Bp_E(R)p_L(R)p_L(T)p_p(R)/(\tau_E + \tau_L(T) + \tau_P) \quad \textbf{(SI\_Equation 2)}$$

In the above expression (**SI_Equation 2**), Parham and Michael 2010 (7) defined *B* to be the number of eggs laid per adult per oviposition, p_E, p_L, and p_P as the daily survival probabilities of eggs, larvae and pupae and τ_E, τ_L, and τ_P as the durations of each of these stages. They assumed *B* to be independent of environmental conditions, development times in each stage to be dependent on temperature only if there was sufficient rainfall to sustain development, and independent effects of temperature and rainfall on the daily survival probability of larvae.

Later in (8) and subsequent work (9-13), the dependence of mosquito density (*M*) on rainfall was dropped, because this relationship is likely to be context-dependent; all time periods were expressed as daily rates; and fecundity in (7) was interpreted to represent total egg production of an individual female adult (*B*) as opposed to the number of eggs laid per adult per oviposition. Further, as data characterizing daily survival probabilities and development times across temperature for each immature stage are scarce, the products of the daily survival probability and the sum of the development times for each stage were replaced with composite traits (p_{EA} and τ_{EA}) representing the entire process. Thus, *EIP* is replaced with the reciprocal of the pathogen development rate (*PDR*), and τ_{EA} is replaced with the reciprocal of the mosquito development rate (*MDR*). Further, as data for *B* were unavailable, *B* was substituted with the expression; $B = EFD/\mu$, where *EFD* is the daily egg production per female and was made to be temperature-dependent (**SI_Equation 3**, **SI_Equation 4**).

$$M(T) = \frac{\lambda(T)}{\mu(T)}\ ;\ \lambda(T) = \frac{B(T)p_{EA}(T)}{\tau_{EA}(T)} = B(T)p_{EA}(T)MDR(T) = \frac{EFD(T)p_{EA}(T)MDR(T)}{\mu(T)} \quad \textbf{(SI\_Equation 3)}$$

SI_Equation 4 is the same formulation used for $R_0(T)_{\text{estimated}}$ (**Eq. 1**) in the main text. In **Eq. 1** *a*, μ, and *EFD* are marked with an * to denote that the data used to parameterize these traits are estimated (as is commonly done in these models) and do not represent the definitions stated above. For example, biting rate, *a*,

is often approximated by using the inverse of the time to the first oviposition instead of directly measuring the number of bites an individual takes in a defined time period.

$$M = \frac{EFD^*(T)pEA(T)MDR(T)}{\mu^*(T)^2} \; ; \; R_0 = \sqrt{\frac{Ma^*(T)^2 bc(T)e^{-\mu/PDR(T)}}{Nr\mu^*(T)}}$$

$$\text{Simplified} \quad R_0 = \sqrt{\frac{EFD^*(T)pEA(T)MDR(T)a^*(T)^2 bc(T)e^{-\mu/PDR(T)}}{Nr\mu^*(T)^3}} \qquad \textbf{(SI\_Equation 4)}$$

To derive the expression for $R_0(T)_{\text{lifetime}}$ (**Eq. 2**) in the main text, we first represented all μ terms with lifespan (*lf*; 1/ μ). Next, as we directly measured *lf*, biting rate (*a*), and the total egg production of an individual female (*B*), the * from these parameters was removed in the expression, and *B* was back substituted in place of *EFD*/μ. Finally, to account for the effects of age-variable mortality rates in the proportion of mosquitoes surviving the latency period, Υ is substituted for exp[-$\mu(T)/PDR(T)$]. The temperature-trait relationship for the proportion of mosquitoes surviving the latency period, $\Upsilon(T)$, is calculated from the Bayesian fit of the proportion of mosquitoes alive (taken from the Gompertz fits to survivorship from each experimental replicate) upon completion of the predicted extrinsic incubation period ($PDR_{50}(T)^{-1}$ or the amount of days to reach 50% of maximum infectiousness in a mosquito population) of *P. falciparum* at each temperature (14).

$$R_0(T)_{lifetime} = \sqrt{\frac{a(T)^2 bc(T)\Upsilon(T)B(T)pEA(T)MDR(T)lf(T)^2}{Nr}} \qquad \textbf{(Eq. 2)}$$

As we do not include values for host-specific traits such as *N* or *r,* and assume these traits are temperature-independent, our static R_0 expression is a relative metric of temperature suitability for transmission as opposed to the traditional interpretation of R_0 as a metric of disease invasion into a fully susceptible population. Further, absolute values of R_0 additionally depend on location-specific factors such as breeding habitat availability, vector biting preference, host availability, disease control efforts, intra- and inter- species interactions, along with additional abiotic factors. Thus, the relative R_0 framework is adopted, and the relationship between relative R_0 and temperature is used to evaluate the impact of model parameterization on the thermal suitability for *P. falciparum* transmission by *An. stephensi* mosquitoes.

Fitting thermal responses in a Bayesian framework

To predict the thermal limits (T_{min}, T_{max}) and optimum (T_{opt}) for each parameter, we used Bayesian inference to fit either a symmetric (quadratic; $-c(T-T_{min})(T-T_{max})$) or an asymmetric (Briere; $cT(T-T_{min})(T_{max}-T)^{1/2}$) unimodal non-linear function to each trait versus temperature (*T, in degrees Celsius*) as in Johnson et al. 2015 (10). Note the parameter *c* is a fit parameter that controls the shape of each respective function. These functions were further restricted to be non-negative. That is, all traits are assumed to be zero if $T < T_{min}$ or $T > T_{max}$). We assumed that data are distributed as truncated normal distributions with the means for each block and temperature described by either the quadratic or Briere function as above. We selected the best-fitting functional form for the mean between quadratic or Briere using the Deviance Information Criterion (DIC) (15)(**SI_Table 6**). We chose to fit thermal responses to the data means across individuals for each replicate as opposed to the raw individual data due to 1) the data exhibiting extreme non-normality for some traits (e.g., lifetime egg production, estimated daily eggs, and lifespan) and thus 2) to ensure compliance with the central limit theorem (CLT) when fitting truncated normal distributions. Developing methodology to account for the non-normal distributions associated with observing individual level data is a key research gap to refine predictions of thermal suitability of transmission events and is an area of future work.

For each parameter in the mean function (i.e., *c*, T_{min}, T_{max}) and the variance of the truncated normal distribution, we assumed relatively uninformative uniform priors that restrict the range of parameters to biologically meaningful values. More specifically, we first fit curves with uninformative priors restricted to biologically informed ranges (T_0 ~ uniform (0, 24), T_m ~ uniform (25, 45), *c* ~ uniform (0, 1)) , followed by informative priors derived from traits estimated in a previous study (10) (assuming a gamma distribution over each component trait). A direct comparison of each temperature-trait response using either uninformative or informative priors is provided to illustrate the influence of informative priors on our trait fits presented in the main text (**SI_Figure 1**). No fit with informative priors was conducted for lifetime egg production (*B*) (**SI_Figure 1C**) as Johnson et al. 2015 (10) did not fit this trait and thus appropriate priors did not exist. Further, we choose to use the fit using uninformative priors for estimated daily eggs (*EFD**) as informative priors altered the thermal response outside the observed data and drastically increased the credible intervals.

This is likely associated with the large uncertainty associated with the prior fit observed in Johnson et al. 2015 (9, 10).

Models were fitted in R using JAGS/rjag (16, 17) , which implements Markov Chain Monte Carlo (MCMC). For details of the specific algorithm see (16) (17). For each thermal trait, posterior draws for the parameters were obtained from three concurrent Moarkv chains. In each chain, a 5,000-step burn-in phase was followed by 20,000 samples of the stationary chain, for a total of 60,000 posterior samples. These samples were then thinned by saving every eighth sample, in order to further reduce autocorrelation in the chain and to reduce computation in the following analyses.

We also defined temperature-trait responses for mosquito and parasite traits not directly measured in this study to assess the impact incorporating multiple trait thermal responses from a single mosquito species (*An. stephensi*), rather than aggregated from several different mosquito species, has on relative $R_0(T)$. *An. stephensi* data from (18) and (14) were used to construct temperature-trait relationships for mosquito development rate (*MDR*), probability of egg to adult survival (*pEA*), *P. falciparum* development rate (*PDR*) and vector competence (*bc*) (**SI_Figure 1G-J**). In contrast, for the Multi-species estimated model we used the thermal relationships defined in (10).

Mapping seasonal transmission range

We generated maps depicting the number of months an area is predicted to be thermally suitable for transmission of human malaria (*P. falciparum*) to illustrate the potential impact differences in the thermal breadth among our relative $R_0(T)$ models have across a relevant landscape. We were primarily interested in comparing the area predicted to be thermally suitable for *P. falciparum* transmission year-round between our two *An. stephensi* relative $R_0(T)$ models incorporating either estimated or observed lifetime trait values across the current distribution of *An. stephensi*. However, we also draw comparisons between our *An. stephensi* models (*An. stephensi* estimated and lifetime) and a previously derived relative $R_0(T)$ model (Multi-species estimated), which aggregates trait data from multiple mosquito and parasite species intended to describe *P. falciparum* transmission in the *An. gambiae* system. This latter comparison serves to illustrate the potential differences in temperature suitability among vectors. Using the posterior median model output, we calculated $R_0(T)$ values at

0.2˚C increments, at a 0.01 level accuracy of model output, rescaled the $R_0(T)$ values from 0-1, and plotted transmission suitability where $R_0(T) > 0$ as in Tesla et al. 2018 (11). Using the GADM global administrative boundaries data we estimated the land area with year-round thermal suitability within countries that span the current range for *An. stephensi*: India, Pakistan, Sri Lanka, Qatar, United Arab Emirates, and Oman (19).

Sensitivity and uncertainty analyses on An. stephensi *$R_0(T)$ models*

To determine if $R_0(T)$ formulation (**Eq. 1** versus **Eq. 2**) affected the sensitivity and uncertainty of R_0 to trait parameters, we performed two types of sensitivity analyses and an uncertainty analysis on our two *An. stephensi* $R_0(T)$ models. For a similar analysis on the Multi-species estimated model see (10). Specifically, our *An. stephensi* lifetime model contains one less μ term due to the substitution of lifetime egg production (B) for EFD/μ and allows age-dependent daily mortality in the proportion of mosquitoes surviving the latency period (Υ). First, to illustrate the degree to which a small change in trait x affects R_0 at a given temperature (T), a model derivative (dR_0/dx) was divided by R_0 for each trait x, to give $dR_0/(R_0\,dx)$ or the standardized sensitivity of R_0 to trait x across all temperatures. Second, to demonstrate the impact of temperature sensitivity of a given trait x on R_0, relative $R_0(T)$ was calculated with each trait held at a constant value and allowing the other parameters to vary with temperature. Finally, we estimated the uncertainty in R_0 introduced through uncertainty in each temperature-trait relationship. To do this we calculated $R_0(T)$ by allowing each trait x to assume its full posterior distribution $x(T)$ while setting all other traits to their posterior median thermal responses. Then, we calculated the width of the 95% credible interval on R_0 at each temperature to estimate the partial uncertainty with respect to x. To determine the full uncertainty in relative $R_0(T)$, the width of the 95% credible interval of R_0 at each temperature was calculated by allowing all parameters to assume their full posterior distribution. We then divided partial uncertainty for each trait by full uncertainty to estimate the proportion of total uncertainty in relative R_0 that is driven by each trait x at each temperature T.

Results

Model formulation affects the sensitivity and uncertainty of relative $R_0(T)$ *to trait parameters*

We conducted two types of sensitivity and an uncertainty analysis to determine if the differences in $R_0(T)$ formulations between the *An. stephensi* lifetime and *An. stephensi* estimated models affected the relative sensitivity and uncertainty of R_0 to different trait parameters. Differences in model formulation did alter the thermal sensitivity of R_0 to each trait parameter as well as which traits contributed most to uncertainty in R_0 across the thermal spectrum (**SI_Figure 2, SI_Figure 3**). However, direct comparison of the relative sensitivity and uncertainty of a given trait between R_0 models should be interpreted cautiously, as traits are not equally represented between models. $R_0(T)$ was sensitive to lifespan (*lf*) and biting rate (*a*) in both *An. stephensi* models; however, the *An. stephensi* lifetime model exhibited less sensitivity to lifespan (*lf*) than the *An. stephensi* estimated model (*An. stephensi* lifetime; **SI_Figure 2B,C** & *An. stephensi* estimated; **SI_Figure 2B,C**). Much of our *An. stephensi* lifetime model uncertainty was attributed to lifetime egg production (*B*, across all temperatures), followed by vector competence (*bc*; cool temperatures), and lifespan (*lf*, warm temperatures) (**SI_Figure 2D**). In contrast, uncertainty around the temperature-trait relationship that contributed the largest proportion of uncertainty in the *An. stephensi* estimated model was from estimated (*lf**) at temperatures greater than 25°C and estimated biting rate (*a**, at intermediate to cooler temperatures), (**SI_Figure 3D**). Estimated daily fecundity (*EFD**, across all temperatures below 30 ^{o}C) also contributed to a fair amount of uncertainty in our *An. stephensi* estimated model (**SI_Figure 3D)**.

Discussion

Study limitations

Several methodological choices made throughout this study likely influenced the outcome of this experiment. First, we measured mosquito life history traits at constant temperatures. Mosquitoes and their pathogens live in thermally fluctuating environments and life history trait values derived at constant temperatures often differ from those measured under temperature fluctuation (18, 20, 21). However, it is not feasible to directly measure trait performance for all possible permutations of temperature fluctuations a mosquito could encounter, while the characterization of temperature-trait responses at constant temperatures is tractable. Second, all larvae were reared at the same standard temperature (27°C) and not at the temperature

adult females were eventually held in order to meet the data inclusion criteria outlined in Mordecai et al 2013 (8, 10). These inclusion criteria were adopted to isolate the effects of temperature on adult mosquito traits from carry-over effects of larval rearing temperature. Yet, numerous studies across different mosquito genera have shown the importance of carry-over effects of larval environments on aspects of adult life history (e.g., body size, survival, reproduction, biting rates, and vector competence) (22). Third, adult mosquitoes were not provided sugar during this experiment. Nutrition treatments can affect adult female life history such as biting rate, fecundity, and survival (23-26). However, the extent of sugar feeding of *An. stephensi* in highly urbanized centers is unknown. Fourth, these experiments were conducted with a laboratory strain of *An. stephensi* due to the logistical difficulties of acquiring field-based *An. stephensi* from malaria endemic regions. Evidence from other ectotherm and dipteran systems, including mosquitoes, suggests thermal adaptation does occur to local environments (27-31). In addition to the possibility of local adaptation, there is also substantial evidence across different mosquito genera that the ability to acquire and transmit pathogens can also vary across mosquito populations (32-34). Thus, wild populations of *An. stephensi* could have different temperature-trait responses and predicted environmental suitability for *P. falciparum* transmission than characterized in this study (35, 36). However, limited evidence comparing thermal performance in long standing colonies to recently field derived colonies suggests the thermal performance characterized from laboratory colonies might be reasonable reflections of thermal performance in the field for some mosquito species, though more work is needed across a diversity of systems to confirm this (36). For example, a recent study found mosquitoes from the wild and a laboratory colony of *An. stephensi* to have similar *Plasmodium vivax* infection rates (37), although this study did not compare infection rates across different temperatures. Finally, in our current study we measure trait data from uninfected adult *An. stephensi* mosquitoes. It is difficult to comment on how our data will vary from infected mosquitoes in the field as the presence or absence of life history trade-offs in *Plasmodium* infected Anopheline mosquitoes is controversial, and the interaction of trait responses (i.e., vector competence, biting rates, lifetime egg production) with temperature and age of exposure have not yet been characterized.

Tables

Supplementary Table 1: Generalized linear mixed models (GLMMs) for proportion imbibed blood

Proportion imbibed blood													
**Model**	**(Intercept)**	**Tscale**	**Dscale**	**Dscale: Tscale**	**I(Dscale^2)**	**I(Tscale^2)**	**I(Dscale^3)**	**I(Dscale^3) : Tscale**	**I(Dscale^3): I(Tscale^2)**	**df**	**LogLik**	**AICc**	**ΔAICc**
m17	***-0.399***	***0.450***	***0.276***	***NA***	***NA***	***-0.203***	***-0.133***	***NA***	***-0.052***	***7***	***-1612.83***	***3239.845***	***NA***
m13	-0.436	0.437	0.296	-0.071	NA	-0.159	-0.183	NA	NA	7	-1616.97	3248.127	8.282
m16	-0.437	0.464	0.303	NA	NA	-0.145	-0.184	-0.019	NA	7	-1618.52	3251.215	11.37
m20	-0.417	0.423	NA	NA	NA	-0.224	-0.026	NA	-0.059	6	-1621.16	3254.45	14.605
m12	-0.513	0.452	-0.025	-0.041	0.060	-0.154	NA	NA	NA	7	-1633.98	3282.152	42.307
m15	-0.548	0.499	0.356	NA	NA	NA	-0.181	0.021	NA	6	-1636.96	3286.055	46.21
m14	-0.548	0.498	0.358	0.030	NA	NA	-0.183	NA	NA	6	-1637.44	3287.024	47.179
m11	-0.634	0.514	0.0415	0.061	0.074	NA	NA	NA	NA	6	-1653.12	3318.366	78.521
m6	-0.579	0.498	0.0184	0.035	NA	NA	NA	NA	NA	5	-1656.13	3322.349	82.504
m4	-0.585	0.495	0.012	0.026	NA	NA	NA	NA	NA	6	-1655.89	3323.91	84.065
m1	-0.608	0.468	NA	NA	NA	NA	NA	NA	NA	3	-1665.59	3337.212	97.367
m3	-0.583	0.486	0.051	0.010	NA	NA	NA	NA	NA	5	-1663.62	3337.329	97.484
m5	-0.583	0.486	0.051	0.010	NA	NA	NA	NA	NA	5	-1663.62	3337.329	97.484
m9	-0.583	0.455	0.051	NA	NA	NA	NA	NA	NA	3	-1671.26	3348.558	108.713
m10	-0.571	0.483	0.0582	0.039	NA	NA	NA	NA	NA	4	-1670.3	3348.67	108.825
m7	-0.604	0.441	NA	NA	NA	NA	NA	NA	NA	2	-1673.39	3350.794	110.949
m18	-0.263	NA	NA	NA	NA	-0.334	-0.047	NA	-0.133	5	-1742.39	3494.874	255.029
m19	-0.262	NA	0.006	NA	NA	-0.334	-0.049	NA	-0.133	6	-1742.38	3496.905	257.06
m2	-0.444	NA	-0.059	NA	NA	NA	NA	NA	NA	3	-1839.31	3684.655	444.81
m8	-0.646	NA	-0.095	NA	NA	NA	NA	NA	NA	2	-1891.59	3787.205	547.36

List of all GLMM models compared for selection of best-fitting values as determined by minimum corrected Akaike Information Criterion (AICc) and log-likelihood (LogLik) values generated in R. Models are ordered from best-fit to worst fit in table. For each fixed effect term included in the model the coefficient supplied by R is listed, if ‘NA” than that term was not included in the model.

Supplementary Table 2: Generalized linear mixed models (GLMMs) for daily egg production

Daily egg production														
**Model**	**(Intercept)**	**Dscale**	**Tscale**	**Dscale : Tscale**	**I(Dscale^2)**	**I(Tscale^2)**	**I(Dscale^2) : I(Tscale^2)**	**I(Dscale^3)**	**I(Dscale^3) : I(Tscale^2)**	**I(Dscale^2): Tscale**	**df**	**LogLik**	**AICc**	**ΔAICc**
m19	***2.673***	***0.327***	***0.369***	***-0.324***	***-0.502***	***-0.240***	***NA***	***NA***	***NA***	***NA***	***8***	***-1532.94***	***3082.113***	***NA***
m13	2.624	0.312	0.366	-0.351	-0.456	-0.187	-0.057	NA	NA	NA	9	-1532.779	3083.85	1.737
m14	2.469	0.368	0.395	-0.200	-0.484	NA	NA	NA	NA	NA	7	-1537.196	3088.573	6.46
m18	2.469	0.368	0.395	-0.200	-0.484	NA	NA	NA	NA	NA	7	-1537.196	3088.573	6.46
m10	2.465	0.362	0.421	NA	-0.408	NA	NA	NA	NA	NA	6	-1540.273	3092.681	10.568
m17	2.465	0.362	0.421	NA	-0.408	NA	NA	NA	NA	NA	6	-1540.273	3092.681	10.568
m12	2.531	0.348	0.422	NA	-0.394	-0.083	NA	NA	NA	NA	7	-1539.577	3093.335	11.222
m22	2.645	0.356	0.418	NA	-0.498	-0.206	0.107	NA	NA	NA	8	-1538.847	3093.926	11.813
m24	2.528	0.364	0.382	NA	-0.386	-0.085	NA	NA	NA	0.044	8	-1539.441	3095.115	13.002
m21	2.235	0.343	0.461	NA	NA	-0.091	NA	-0.160	0.096	NA	8	-1548.162	3112.557	30.444
m15	2.259	0.180	0.361	-0.162	NA	-0.209	NA	NA	NA	NA	7	-1549.189	3112.559	30.446
m11	2.237	0.209	0.387	NA	NA	-0.134	NA	NA	NA	NA	6	-1550.62	3113.376	31.263
m20	2.280	0.316	0.360	-0.169	NA	-0.213	NA	-0.088	NA	NA	8	-1548.693	3113.619	31.506
m8	2.112	0.226	0.390	NA	NA	NA	NA	NA	NA	NA	5	-1552.301	3114.699	32.586
m16	2.126	0.335	0.391	NA	NA	NA	NA	-0.069	NA	NA	6	-1551.989	3116.114	34.001
m4	2.098	0.222	0.385	-0.044	NA	NA	NA	NA	NA	NA	6	-1552.173	3116.481	34.368
m5	2.098	0.222	0.385	-0.044	NA	NA	NA	NA	NA	NA	6	-1552.173	3116.481	34.368
m9	2.098	0.222	0.385	-0.044	NA	NA	NA	NA	NA	NA	6	-1552.173	3116.481	34.368
m1	2.098	0.222	0.385	-0.044	NA	NA	NA	NA	NA	NA	7	-1552.173	3118.527	36.414
m2	2.128	NA	0.308	NA	NA	NA	NA	NA	NA	NA	4	-1555.895	3119.854	37.741
m6	2.128	NA	0.308	NA	NA	NA	NA	NA	NA	NA	4	-1555.895	3119.854	37.741
m23	2.731	NA	NA	NA	-0.465	-0.267	0.128	NA	NA	NA	6	-1556.472	3125.079	42.966
m7	2.167	0.057	NA	NA	NA	NA	NA	NA	NA	NA	4	-1564.466	3136.996	54.883
m3	2.167	0.057	NA	NA	NA	NA	NA	NA	NA	NA	4	-1564.466	3136.996	54.883

List of all GLMM models compared for selection of best-fitting values as determined by minimum corrected Akaike Information Criterion (AICc) and log-likelihood (LogLik) values generated in R. Models are ordered from best-fit to worst fit in table. For each fixed effect term included in the model the coefficient supplied by R is listed, if ‘NA” than that term was not included in the model.

Supplemental Table 3: Temperature and day effects on trait values

Trait	Model type	Random Effects	Family	Fixed Effects	Coef.	χ^2	df	p
Proportion imbibed blood	GLMM	Female	Binomial (link = 'logit')	(Intercept)	-0.399			
				Temperature	0.450	267.882	1	*<0.001*
				Day	0.276	16.915	1	*<0.001*
				Temperature^2	-0.203	34.840	1	*<0.001*
				Day^3	-0.133	35.202	1	*<0.001*
				Tempature^2 *Day^3	-0.052	13.293	1	*<0.001*
Daily egg production	GLMM	Donor	Gamma (link = 'log')	(Intercept)	2.673			
				Temperature	0.369	38.379	1	*<0.001*
				Day	0.327	20.046	1	*<0.001*
				Temperature *Day	-0.324	14.532	1	*<0.001*
				Temperature^2	-0.240	8.911	1	0.003
				Day^2	-0.502	35.725	1	*<0.001*
Survivorship	Survival	Block	Log-rank test	Temperature		220	5	<0.001
	Distribution	Exponential	Gamma	Gompertz	Log-normal	Weibull		
	AIC	2979	2816	2758	2913	2773		

Supplemental Table 4: Survival distribution AIC values by temperature treatment

Temperature	Survival Distribution				
	Exponential	Gamma	***Gompertz***	Log-normal	Weibull
16	453	437	***431***	451	433
20	451	428	***418***	439	422
24	487	438	438	441	***436***
28	759	720	***693***	746	708
32	447	437	***426***	456	432
36	380	346	***325***	360	336

Supplemental Table 5: Source of data included in relative $R_0(T)$ models

Model	Parameter Source		
	a, B, lf	*bc, PDR*	*MDR, pEA*
An. stephensi[a]	**this study**	*Shapiro et al. 2017*	*Paaijmans et al. 2013*
multi-species[b]	*Johnson et al. 2015*	*Johnson et al. 2015*	*Johnson et al. 2015*

Parameters: daily biting rate (*a*), lifetime egg production (*B*), lifespan (*lf*), vector competence (*bc*), parasite development rate (*PDR*), mosquito development rate (*MDR*), and the probability of egg to adult survival (*pEA*).[a]denotes $R_0(T)$ models describing the thermal suitability of transmission of *P. falciparum* via *An. stephensi*. [b]indicates the previous $R_0(T)$ model intended to describe the thermal suitability of the transmission of *P. falciparum* via *An. gambiae*.

Supplemental Table 6: Functional forms of thermal responses

Trait	Function	DIC[a]	Parameters[b]		
			c	T_{min}	T_{max}
biting rate (a)	**Briere**<br>quadratic	**-48.794**<br>-16.295	**0.000175776**	**3.490979**	**41.8226**
estimated biting rate (a^*)	**Briere**<br>quadratic	**-50.941**<br>-32.078	**0.00009909**	**11.75336**	**43.94362**
lifespan (lf)	Briere<br>**quadratic**	100.235<br>**84.382**	**0.1210401**	**1.9334458**	**37.5509388**
estimated lifespan (lf^*)	Briere<br>**quadratic**	82.377<br>**60.470**	**0.05050002**	**1.73644511**	**37.59197227**
lifetime egg production (B)	**Briere**<br>quadratic	**163.676**<br>168.165	**0.3335789**	**8.9871276**	**32.9394485**
estimated daily eggs (EFD^*)	**Briere**<br>quadratic	**72.796**<br>84.474	**0.007258325**	**8.577127443**	**39.99476119**
pathogen development rate (PDR)	**Briere**<br>quadratic	**-79.868**<br>-2.843	**0.000054155**	**9.064107**	**43.51257**
vector competence (bc)	Briere<br>**quadratic**	5.308<br>**-18.244**	**0.003672395**	**12.11497854**	**38.13451902**
prob. egg to adult survival (p_{EA})	Briere<br>**quadratic**	-73.029<br>**-63.677**	**0.00809132**	**15.35285981**	**36.95293814**
mosq. development rate (MDR)	**Briere**<br>quadratic	**-435.733**<br>-300.965	**0.000106632**	**13.36284**	**35.96712**
gamma (Υ)	Briere<br>**quadratic**	10.859<br>**-21.082**	**0.00317107**	**8.75713349**	**43.22849889**

[a]Deviance Information Criterian (DIC) were used to select the best-fitting functional form, where a more negative value represents a better fit. Model fits were assessed on the fits using uniformative priors. Quadratic function; $-c(T-T_{min})(T-T_{max})$ or Briere function; $cT(T-T_{min})(T_{max}-T)^{1/2}$ with T representing temperature in degrees Celsius. [b]Parameter values are based on means of MCMC simulations. Functional forms and parameters used in the relative R_0 expression are bolded.

Supplemental Table 7: Thermal thresholds of temperature-trait relationships for lifetime values and estimates

Trait	Lifetime Values	Function	Thermal threshold (95% CI[a])			
			T_{min} (°C)	T_{opt} (°C)	T_{max} (°C)	$T_{breadth}$ (°C)[b]
biting rate	observed (a)	Briere	3.2 (0.2-6.6)	33.8 (32.2-35.4)	41.8 (39.8-44)	38.6 (33.2-43.8)
	estimated (a*)		11.6 (5.6-17.2)	36.4 (33.2-40)	43 (40-45)	31.4 (22.8-39.4)
lifespan	observed (lf)	quadratic	1.6 (0-4.6)	19.6 (18.4-21.4)	37.6 (35.4-39.6)	36 (30.8-39.6)
	estimated (lf*)		1.4 (0-4.4)	19.6 (18.2-21)	37.8 (35.8-39.6)	31.4 (30.8-39.6)
lifetime egg production	observed (B)	Briere	8.8 (0-17.2)	27.6 (24-30)	33.2 (28.4-36)	24.4 (11.2-36)
	estimated (B*)	Product[c]	8.4 (0-16.2)	26.8 (25.2-28.6)	39.8 (36.8-44.4)	31.4 (20.6-44.4)

Thermal threshold values are based on median model outputs. [a]CI represents the credible interval of Bayesian fits. [b]$T_{breadth}$ is the range of temperatures that the trait-function is greater than 0 (T_{max}-T_{min}). [c]Product references that estimated lifetime egg production (B*) was not directly fit to data, but rather is the product of the temperature-trait relations for *EFD** (Briere function) and *lf** (Quadratic function). Data source is from the lifetable experiment conducted in this study.

Supplemental Table 8: Thermal thresholds of relative $R_0(T)$ models

$R_0(T)$ model	Thermal threshold (95% CI)			
	T_{min} (°C)	T_{opt} (°C)	T_{max} (°C)	$T_{breadth}$ (°C)[b]
An. stephensi lifetime	15.6 (14.8-18.6)	27 (26.8-27)	33 (28.6-35.8)	17.4 (10-21)
An. stephensi estimated	15.6 (14.8-19.2)	27.6 (27.4-27.6)	35.8 (34.6-35.8)	20 (15.4-21)
Multi-species estimated	19.2 (16.4-23)	25.6 (24.4-26)	32.4 (29.4-34.2)	13.2 (6.4-17.8)

Thermal threshold values are based on scaled and rounded median model outputs. [a]CI represents the credible interval. [b]$T_{breadth}$ is the range of temperatures that $R_0(T) > 0$ (T_{max}-T_{min}). Thus, any temperature less than the T_{min} or greater than the T_{max} is deemed unsuitable for transmission to occur provided the vector is present.

Supplemental Table 9: Thermal thresholds of *Y(T)* across relative $R_0(T)$ models

Model	Thermal threshold (95% CI)			
	T_{min} (°C)	T_{opt} (°C)	T_{max} (°C)	$T_{breadth}$ (°C)[b]
An. stephensi lifetime	8.8 (5.8-11.2)	26 (24.6-27)	43.4 (41.4-45)	34.6 (30.2-39.2)
An. stephensi estimated	9.7 (9.1-12.9)	28.3 (27.4-29.2)	37.3 (34.6-38)	27.6 (21.7-28.9)
Multi-species estimated	14 (13-15)	24.8 (23.6-26)	34.4 (33.6-34.8)	20.4 (18.6-21.8)

Thermal threshold values are based on scaled median model outputs. [a]CI represents the credible interval. [b]$T_{breadth}$ is the range of temperatures that *Y(T)* > 0 (T_{max}-T_{min}).

Supplemental Table 10: Area thermally suitable for year-round transmission

Country	Year-round temperature suitability Area (km^2)		
	An. stephensi lifetime	*An. stephensi* estimated	Multi-species estimated
India	1,352,222	2,372,906	710,046
Oman	225,632	299,702	103,645
Pakistan	51,244	120,634	547
Qatar	0	11,210	0
Sri Lanka	65,996	65,991	64,299
U.A.E.	4,415	79,856	78

Figures

Supplemental Figure 1. Direct comparison of uniform to informative priors. Direct comparison on how the use of uniform (black) or informative (orange) priors influences the temperature-trait relationship and 95% credible intervals for (**A**) bite rate (*a*), (**B**) lifespan (*lf*), (**C**) lifetime egg production (*B*), (**D**) estimated biting rate (*a**), (**E**) estimated lifespan (*lf**), (**F**) estimated daily egg production (*EFD**), (**G**) vector competence (*bc*), (**H**) parasite development rate (*PDR*), (**I**) probability of egg to adult survival (*pEA*), (**J**) mosquito development rate (*MDR*), (**K**) relative R_0 (*An. stephensi* lifetime model), and (**L**) relative R_0 (*An. stephensi* estimated model). Trait thermal performance curves from Johnson et al. 2015 (10) were used as informative priors. No fit with informative priors was generated for lifetime egg production (*B*) in panel **C** as Johnson et al. 2015 did not conduct a fit with data in this form. The uninformative fit for *EFD** was used in the informative relative R_0 model for panel **L** as priors altered the curve shape away from our dataset and increased the credible intervals. Of note, the credible intervals (faded regions) describe the uncertainty around the mean value of the trait fit at each temperature, and do not represent a predictive interval on the data, which contains additional uncertainty due to the inferred normal distribution around the TPC means.

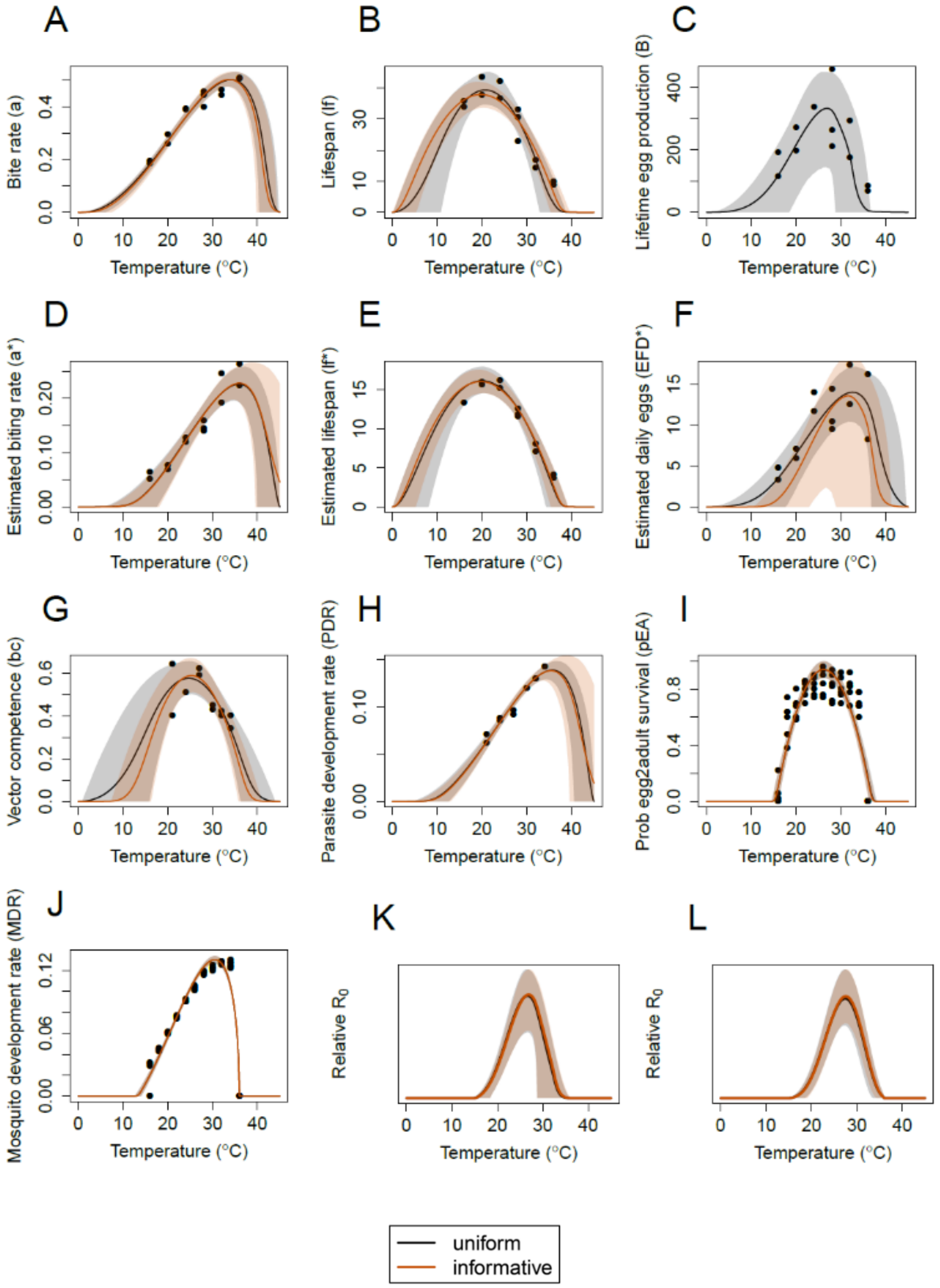

Supplemental Figure 2. Histograms of the posterior distributions for the critical thermal thresholds for each observed and estimated trait. Histograms of the posterior distribution of the Bayesian fit using a 0.4°C bin width of the critical thermal thresholds (T_{min}, T_{opt}, and T_{max}) between observed lifetime traits (black) and estimated lifetime traits (blue) for biting rate (**a**), lifespan (**b**), and lifetime egg production (**c**).

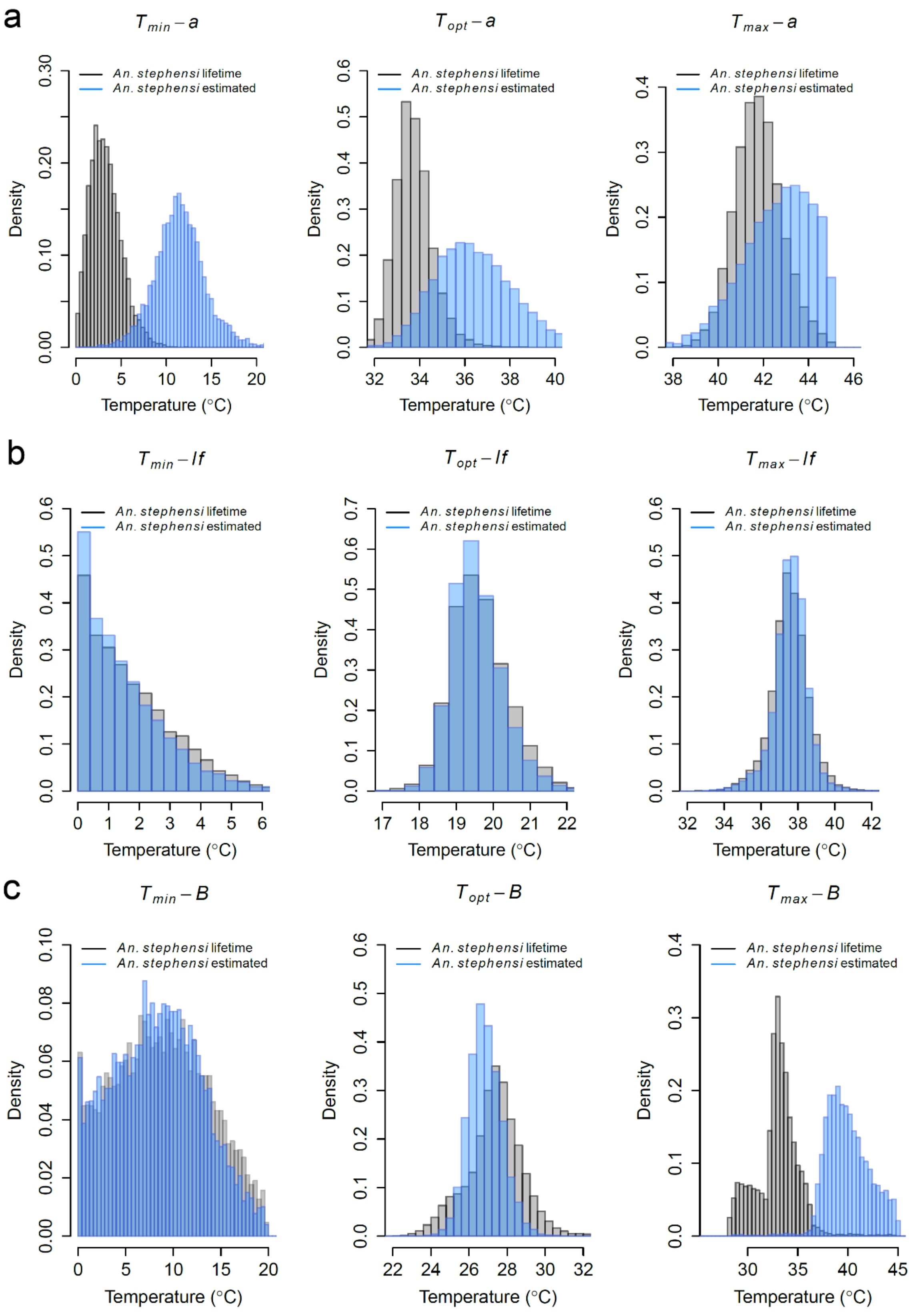

Supplemental Figure 3. Histograms of the posterior distributions for the critical thermal thresholds for each relative R_0 fit. Histograms of the posterior distribution using a 0.4°C bin width for each relative $R_0(T)$ model (Multi-species estimated: purple; *An. stephensi* lifetime: black; *An. stephensi* estimated: blue) across the T_{min} (**a**), T_{opt} (**b**), and T_{max} (**c**) critical thresholds.

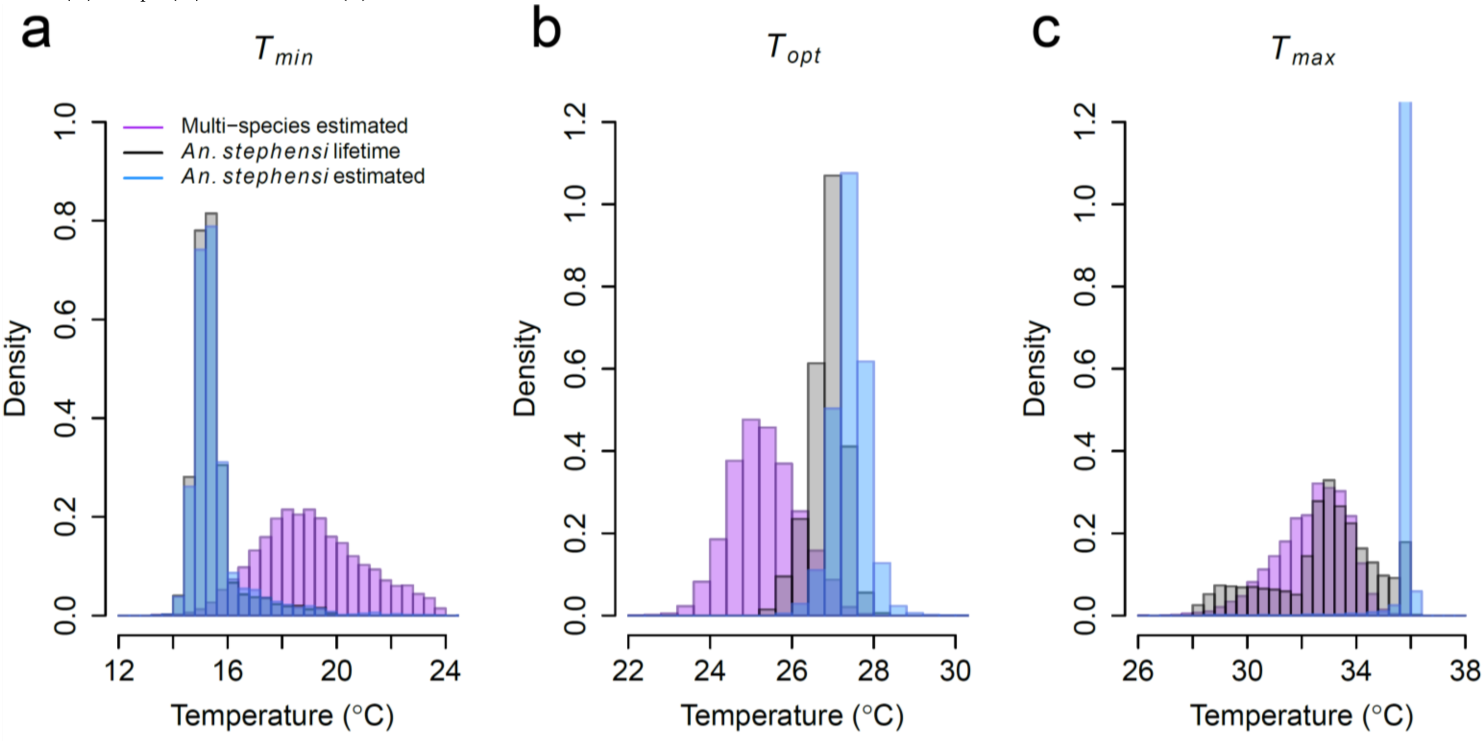

Supplemental Figure 4. Sensitivity and uncertainty analysis on *An. stephensi* lifetime $R_0(T)$ model. Relative $R_0(T)$ *An. stephensi* lifetime model (**A**) with mean model outputs (solid black line) and 95% credible intervals (dashed black lines). Sensitivity analysis based on formula derivatives for *An. stephensi* lifetime $R_0(T)$ where for each trait x, dR_0/dx was divided by R_0, to give dR_0/R_0dx, or the standardized sensitivity of R_0 to a parameter x, across all temperatures (**B**). A second sensitivity analysis based on setting each parameter constant across temperature for *An. stephensi* lifetime where relative $R_0(T)$ was calculated with a single trait held constant and allowing the other parameters to vary with temperature (**C**). Uncertainty analysis on *An. stephensi* lifetime (**D**). For the uncertainty analysis, each trait x was allowed to assume its full posterior distribution $x(T)$, while setting all other traits to their posterior median thermal responses and calculating R_0. Partial uncertainty with respect to trait x is the width of the 95% credible interval on $R_0(T)$ at each temperature. Full uncertainty was calculated by allowing all parameters to assume their full posterior distribution and calculating the width of the 95% credible interval of $R_0(T)$ at each temperature. Partial uncertainty for each trait divided by full uncertainty of $R_0(T)$ gives the proportion of total uncertainty in $R_0(T)$ that is driven by each trait x at each temperature, T.

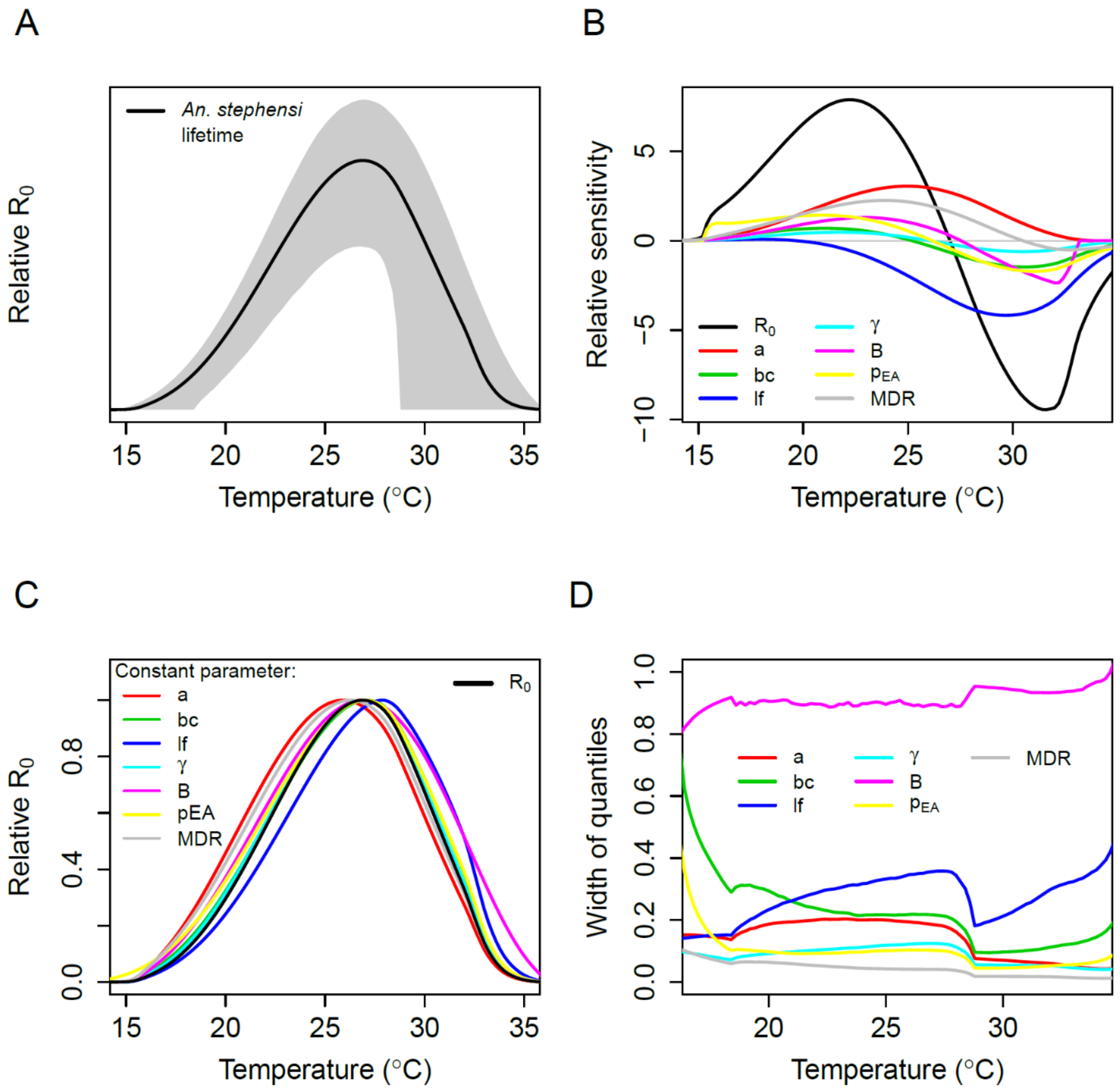

Supplemental Figure 5. Sensitivity and uncertainty analysis on *An. stephensi* estimated $R_0(T)$ model. Relative $R_0(T)$ *An. stephensi* estimated model (**A**) with mean model outputs (solid black line) and 95% credible intervals (dashed black lines). Sensitivity analysis based on formula derivatives for *An. stephensi* estimated $R_0(T)$ where for each trait x, dR_0/dx was divided by R_0, to give dR_0/R_0dx, or the standardized sensitivity of R_0 to a parameter x, across all temperatures (**B**). A second sensitivity analysis based on setting each parameter constant across temperature for *An. stephensi* estimated where relative $R_0(T)$ was calculated with a single trait held constant and allowing the other parameters to vary with temperature (**C**). Uncertainty analysis on *An. stephensi* estimated (**D**). For the uncertainty analysis, each trait x was allowed to assume its full posterior distribution $x(T)$, while setting all other traits to their posterior median thermal responses and calculating R_0. Partial uncertainty with respect to trait x is the width of the 95% credible interval on $R_0(T)$ at each temperature. Full uncertainty was calculated by allowing all parameters to assume their full posterior distribution and calculating the width of the 95% credible interval of $R_0(T)$ at each temperature. Partial uncertainty for each trait divided by full uncertainty of $R_0(T)$ gives the proportion of total uncertainty in $R_0(T)$ that is driven by each trait x at each temperature, T.

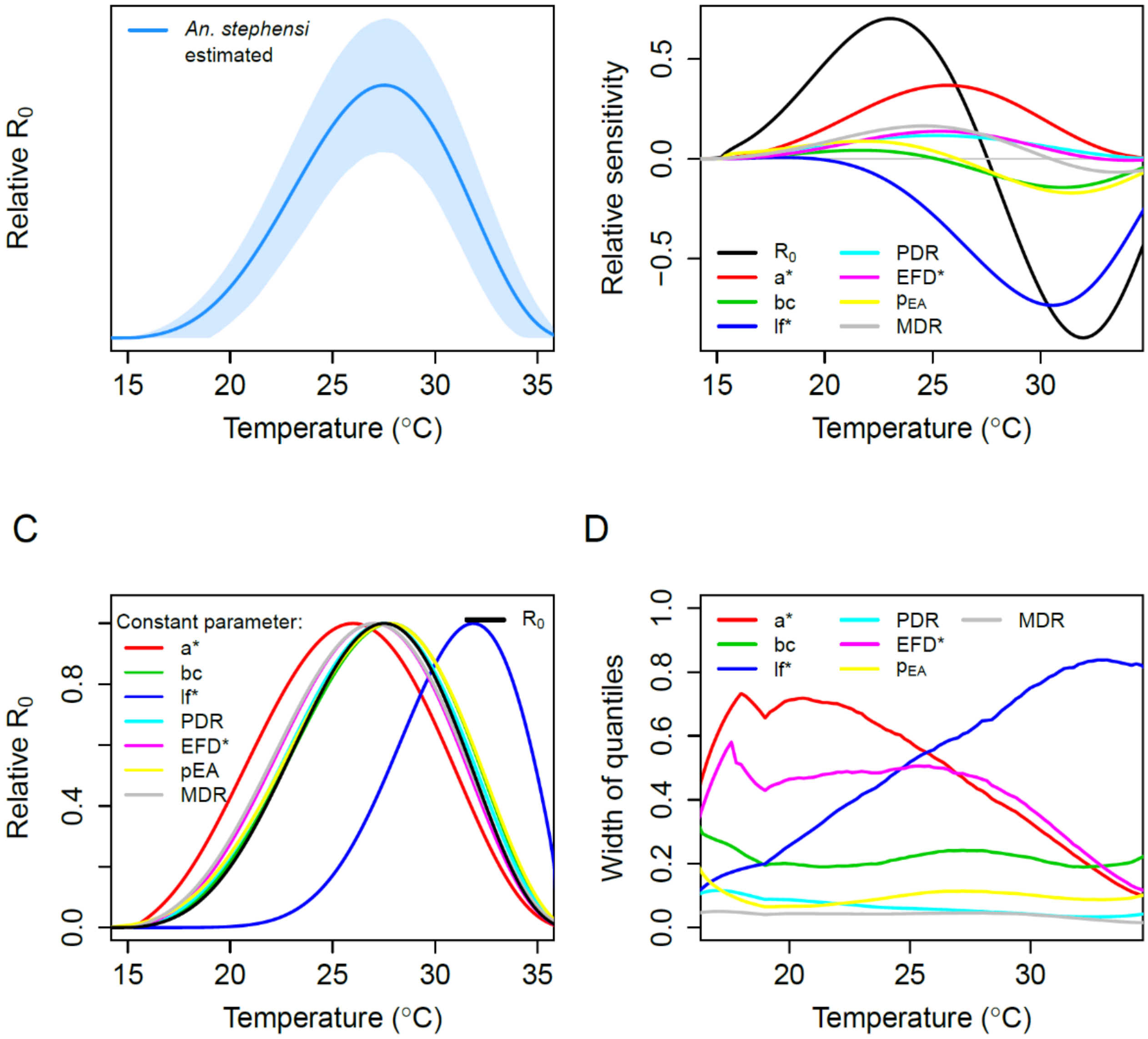

Supplemental Figure 6. Overlay of gamma (Υ), the proportion of mosquitoes surviving past the latency period used in the relative $R_0(T)$ models. The *An. stephensi* lifetime model incorporates the temperature-trait relationship for the parameter gamma, $\Upsilon(T)$, the proportion of mosquitoes surviving the latency period. We generated $\Upsilon(T)$ for the *An. stephensi* lifetime model by fitting a quadratic function with Bayesian inference over the proportion of mosquitoes alive (taken from the Gompertz fits to survivorship from each experimental replicate) upon completion of the predicted extrinsic incubation period ($PDR50(T)^{-1}$) of *P. falciparum* at each temperature (as fit from the EIP_{50} values from (14)). $\Upsilon(T)$ for the *An. stephensi* and multi-species estimated models can be derived indirectly from the following expression: $\Upsilon(T) = \exp[-\mu^*(T)/PDR(T)]$. In the *An. stephensi* estimated model μ^* was calculated by assuming an exponential function over a truncated portion of the Kaplan-Meier survival estimates as specified in (8). In the multi-species estimated model $\Upsilon(T)$ was also indirectly calculated using the expression $\Upsilon(T) = \exp[-\mu^*(T)/PDR(T)]$, however, $\mu^*(T)$ and $PDR(T)$ are from the fits generated in Johnson et al. 2015.

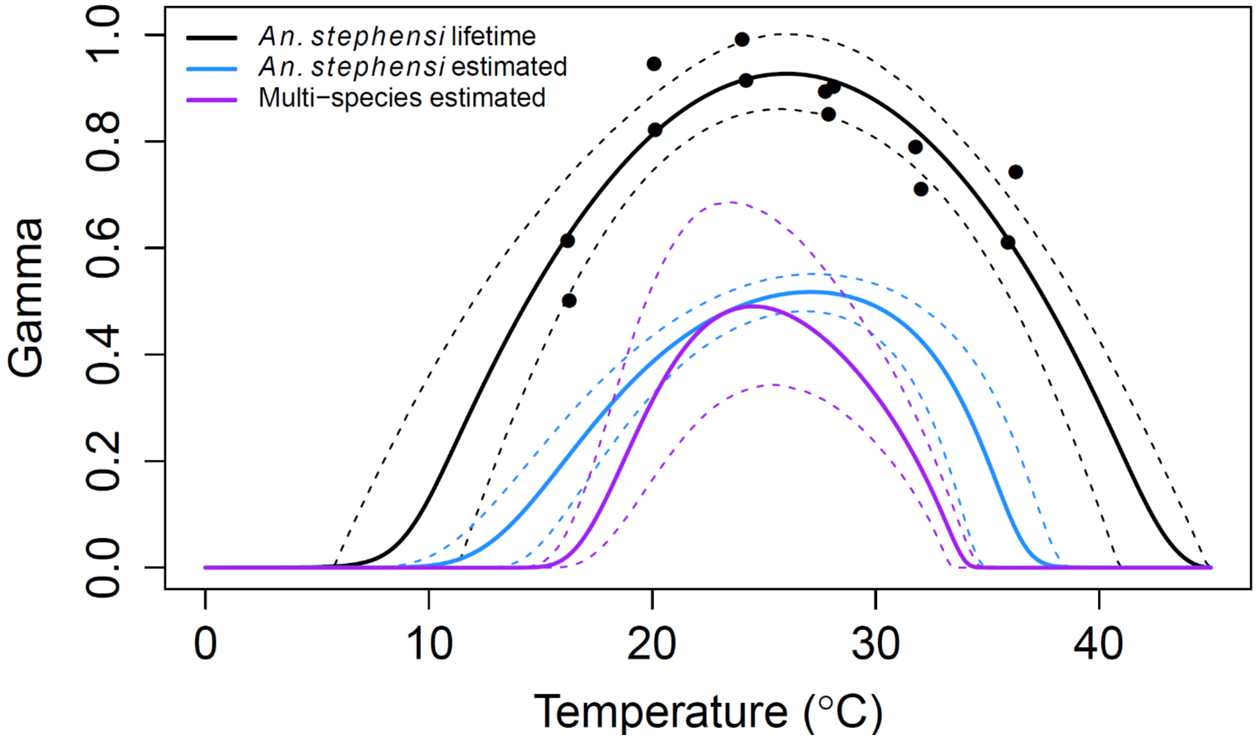

Supplemental Figure 7. Thermal responses included in the *An. stephensi* relative $R_0(T)$ models. Thermal responses included in the *An. stephensi* relative $R_0(T)$ models. (**A**) bite rate (*a*), (**B**) lifespan (*lf*), (**C**) lifetime egg production (*B*), (**D**) estimated biting rate (*a**), (**E**) estimated lifespan (*lf**), (**F**) estimated daily egg production (*EFD**), (**G**) vector competence (*bc*), (**H**) parasite development rate (*PDR*), (**I**) probability of egg to adult survival (*pEA*), and (**J**) mosquito development rate (*MDR*). Traits only included in the *An. stephensi* lifetime model (black; **A-C**); Traits only included in the *An. stephensi* estimated model (blue; **D-F**), or traits included in both *An. stephensi* models (green; **G-J**).

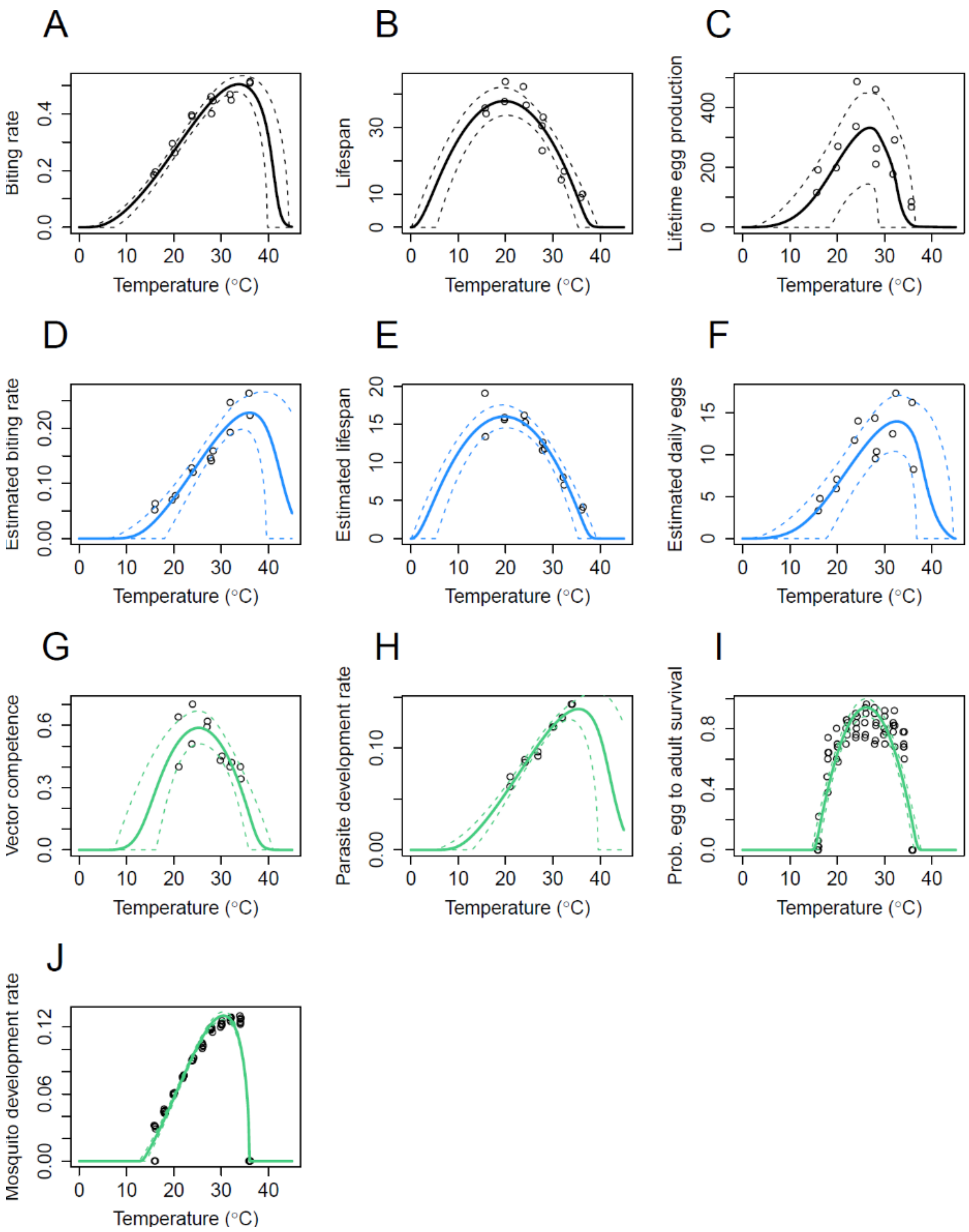

References

1. R Core Team. R: A Language and Environment for Statistical Computing. Vienna, Austria: R Foundation for Statistical Computing; 2017.
2. Bates D, Machler M, Bolker BM, Walker SC. Fitting Linear Mixed-Effects Models Using lme4. J Stat Softw. 2015;67(1):1-48.
3. Sakamoto Y, Ishiguro, M., and Kitagawa G. . Akaike Information Criterion Statistics: D. Reidel Publishing Company; 1986.
4. Therneau TM, Grambsch PM. Modeling survival data : extending the Cox model. New York: Springer; 2000. xiii, 350 p. p.
5. Jackson CH. flexsurv: A Platform for Parametric Survival Modeling in R. J Stat Softw. 2016;70(8):1-33.
6. Dietz K. The estimation of the basic reproduction number for infectious diseases. Stat Methods Med Res. 1993;2(1):23-41.
7. Parham PE, Michael E. Modeling the effects of weather and climate change on malaria transmission. Environ Health Perspect. 2010;118(5):620-6.
8. Mordecai EA, Paaijmans KP, Johnson LR, Balzer C, Ben-Horin T, de Moor E, et al. Optimal temperature for malaria transmission is dramatically lower than previously predicted. Ecol Lett. 2013;16(1):22-30.
9. Mordecai EA, Cohen JM, Evans MV, Gudapati P, Johnson LR, Lippi CA, et al. Detecting the impact of temperature on transmission of Zika, dengue, and chikungunya using mechanistic models. PLoS Negl Trop Dis. 2017;11(4):e0005568.
10. Johnson LR, Ben-Horin T, Lafferty KD, McNally A, Mordecai E, Paaijmans KP, et al. Understanding uncertainty in temperature effects on vector-borne disease: a Bayesian approach. Ecology. 2015;96(1):203-13.
11. Tesla B, Demakovsky LR, Mordecai EA, Ryan SJ, Bonds MH, Ngonghala CN, et al. Temperature drives Zika virus transmission: evidence from empirical and mathematical models. Proc Biol Sci. 2018;285(1884).
12. Mordecai EA, Caldwell JM, Grossman MK, Lippi CA, Johnson LR, Neira M, et al. Thermal biology of mosquito-borne disease. Ecol Lett. 2019.
13. Shocket MS, Ryan SJ, Mordecai EA. Temperature explains broad patterns of Ross River virus transmission. Elife. 2018;7.
14. Shapiro LLM, Whitehead SA, Thomas MB. Quantifying the effects of temperature on mosquito and parasite traits that determine the transmission potential of human malaria. PLoS Biol. 2017;15(10):e2003489.
15. Spiegelhalter DJ, Best, N. G., Carlin, B. P. and Van Der Linde, A. . Bayesian measures of model complexity and fit. Journal of the Royal Statistical Society. 2002(Series B 64):583-639.
16. Plummer M, editor JAGS: A program for analysis of Bayesian graphical models using Gibbs sampling. Proceedings of the 3rd International Workshop on Distributed Statistical Computing; 2003; Vienna, Austria.
17. Plummer M. rjags: Bayesian Graphical Models using MCMC. R package version 4-6. 2016.
18. Paaijmans KP, Heinig RL, Seliga RA, Blanford JI, Blanford S, Murdock CC, et al. Temperature variation makes ectotherms more sensitive to climate change. Glob Chang Biol. 2013;19(8):2373-80.
19. Kiszewski A, Mellinger A, Spielman A, Malaney P, Sachs SE, Sachs J. A global index representing the stability of malaria transmission. Am J Trop Med Hyg. 2004;70(5):486-98.
20. Paaijmans KP, Blanford S, Bell AS, Blanford JI, Read AF, Thomas MB. Influence of climate on malaria transmission depends on daily temperature variation. Proc Natl Acad Sci U S A. 2010;107(34):15135-9.
21. Murdock CC, Sternberg ED, Thomas MB. Malaria transmission potential could be reduced with current and future climate change. Sci Rep. 2016;6:27771.
22. Evans MV, Shiau JC, Solano N, Brindley MA, Drake JM, Murdock CC. Carry-over effects of urban larval environments on the transmission potential of dengue-2 virus. Parasit Vectors. 2018;11(1):426.
23. Okech BA, Gouagna LC, Killeen GF, Knols BG, Kabiru EW, Beier JC, et al. Influence of sugar availability and indoor microclimate on survival of Anopheles gambiae (Diptera: Culicidae) under semifield conditions in western Kenya. J Med Entomol. 2003;40(5):657-63.
24. Gary RE, Jr., Foster WA. Effects of available sugar on the reproductive fitness and vectorial capacity of the malaria vector Anopheles gambiae (Diptera: Culicidae). J Med Entomol. 2001;38(1):22-8.

25. Scott TW, Chow E, Strickman D, Kittayapong P, Wirtz RA, Lorenz LH, et al. Blood-feeding patterns of Aedes aegypti (Diptera: Culicidae) collected in a rural Thai village. J Med Entomol. 1993;30(5):922-7.
26. Scott TW, Naksathit A, Day JF, Kittayapong P, Edman JD. A fitness advantage for Aedes aegypti and the viruses it transmits when females feed only on human blood. Am J Trop Med Hyg. 1997;57(2):235-9.
27. Ruybal JE, Kramer LD, Kilpatrick AM. Geographic variation in the response of Culex pipiens life history traits to temperature. Parasit Vectors. 2016;9:116.
28. Sternberg ED, Thomas MB. Local adaptation to temperature and the implications for vector-borne diseases. Trends Parasitol. 2014;30(3):115-22.
29. Asgharian H, Chang PL, Lysenkov S, Scobeyeva VA, Reisen WK, Nuzhdin SV. Evolutionary genomics of Culex pipiens: global and local adaptations associated with climate, life-history traits and anthropogenic factors. Proc Biol Sci. 2015;282(1810).
30. Kellermann V, Overgaard J, Hoffmann AA, Flojgaard C, Svenning JC, Loeschcke V. Upper thermal limits of Drosophila are linked to species distributions and strongly constrained phylogenetically. Proc Natl Acad Sci U S A. 2012;109(40):16228-33.
31. Lazzaro BP, Flores HA, Lorigan JG, Yourth CP. Genotype-by-environment interactions and adaptation to local temperature affect immunity and fecundity in Drosophila melanogaster. PLoS Pathog. 2008;4(3):e1000025.
32. Reisen WK, Fang Y, Martinez VM. Effects of temperature on the transmission of west nile virus by Culex tarsalis (Diptera: Culicidae). J Med Entomol. 2006;43(2):309-17.
33. Lambrechts L, Halbert J, Durand P, Gouagna LC, Koella JC. Host genotype by parasite genotype interactions underlying the resistance of anopheline mosquitoes to Plasmodium falciparum. Malar J. 2005;4:3.
34. Alout H, Ndam NT, Sandeu MM, Djegbe I, Chandre F, Dabire RK, et al. Insecticide resistance alleles affect vector competence of Anopheles gambiae s.s. for Plasmodium falciparum field isolates. PLoS One. 2013;8(5):e63849.
35. Huho BJ, Ng'habi KR, Killeen GF, Nkwengulila G, Knols BG, Ferguson HM. Nature beats nurture: a case study of the physiological fitness of free-living and laboratory-reared male Anopheles gambiae s.l. J Exp Biol. 2007;210(Pt 16):2939-47.
36. Lyons CL, Coetzee M, Terblanche JS, Chown SL. Thermal limits of wild and laboratory strains of two African malaria vector species, Anopheles arabiensis and Anopheles funestus. Malar J. 2012;11:226.
37. Mohanty AK, Nina PB, Ballav S, Vernekar S, Parkar S, D'Souza M, et al. Susceptibility of wild and colonized Anopheles stephensi to Plasmodium vivax infection. Malar J. 2018;17(1):225.
